# Drugs, cancer and end-of-life care: A case study of pharmaceuticalization?

**DOI:** 10.1016/j.socscimed.2014.12.007

**Published:** 2015-04

**Authors:** Courtney Davis

**Affiliations:** Department of Social Science, Health and Medicine, School of Social Science and Public Policy, King's College London, Strand, London WC2R 2LS, UK

**Keywords:** United States, United Kingdom, Pharmaceuticalization, End-of-life cancer care, Expectations, Overtreatment, Patient preferences, Clinical benefit

## Abstract

There is evidence from some countries of a trend towards increasingly aggressive pharmacological treatment of patients with advanced, incurable cancer. To what extent should this be understood as a progressive development in which technological innovations address previously unmet needs, or is a significant amount of this expansion explained by futile or even harmful treatment? In this article it is argued that while some of this growth may be consistent with a progressive account of medicines consumption, part of the expansion is constituted by the inappropriate and overly aggressive use of drugs. Such use is often explained in terms of individual patient consumerism and/or factors to do with physician behaviour. Whilst acknowledging the role of physicians and patients' expectations, this paper, drawing on empirical research conducted in the US, the EU and the UK, examines the extent to which upstream factors shape expectations and drive pharmaceuticalisation, and explores the value of this concept as an analytical tool.

## Highlights

• Patients are increasingly likely to receive chemotherapy towards the end-of-life.
• Some of this treatment is unnecessary and harmful to patients.
• Clinical benefits do not match many patients' informed preferences.
• Low regulatory standards and misinformation are key drivers of overtreatment.

## 1 Introduction

Since the mid-1990s, a number of studies have focused attention on the growing importance of pharmaceuticals in our day-to-day lives. This work has been reviewed by Abraham (2009, 2010) and by Williams et al. (2011), and recent articles by these scholars and by Busfield (2010) from the United Kingdom (UK) have provided broad overviews of the rapid expansion in medicines use over recent decades, and suggested conceptual and heuristic frameworks for the development of future sociological analyses. Abraham and Williams et al. suggest that processes of ‘pharmaceuticalization’ – ‘[t]he transformation of human conditions, capacities or capabilities into opportunities for pharmaceutical intervention’ (Williams et al., 2011, 711) – have driven this expansion, and all authors agree that the trend cannot be adequately explained by ‘progressive’ accounts of techno-scientific progress meeting population health needs – what Abraham calls the ‘biomedicalism thesis’ (2010, 606).

As Abraham argues, addressing the validity of biomedical explanations is crucial to any analysis since it is directly relevant to sociological understanding and evaluation of the impacts of pharmaceuticalisation, including ‘the implications of pharmaceuticalization for health’ (Abraham, 2010, 606). Of the three overviews, only Abraham attempts in any substantive way to address the plausibility of what he calls the biomedicalism thesis as an explanation for *overall* pharmaceutical expansion, arguing that, given an overall decline in therapeutic innovation since the late 1990s, ‘biomedicalism … cannot be an explanation for the *growth* in overall pharmaceutical markets or *expanded* pharmaceuticalization in some therapeutic areas, because no such growth or expansion of drug innovation offering significant therapeutic advance has occurred in the last 15–20 years’ (2010, 616). However, this argument is problematic in that it fails to recognise that increased rates of medicines prescribing can also be explained by increased utilisation of *existing* drugs to meet the *established* health needs of a growing patient population, despite declining rates of therapeutic innovation. Patient populations may expand due to demographic factors, higher incidence rates of disease, and/or improved diagnosis or access to healthcare. In such cases, biomedical explanations *may* provide a sufficient explanation for higher rates of medicines utilisation. More detailed analyses of patterns of use, the plausibility of different explanatory factors, and the impacts of increased medicines consumption within specific disease areas is needed to test ‘progressive’ explanations and shed further light on the usefulness of ‘pharmaceuticalization’ as a conceptual tool.

This article explores the drivers and impacts of expanding medicines use in the treatment of patients with advanced, metastatic solid tumour cancers in the US and the EU. The analysis draws on research, undertaken between 2008 and 2013, investigating the dynamics of patient advocacy and the regulation of new anticancer drugs. This research involved extensive documentary data collection and analysis, including review of the scientific, social scientific and ‘grey’ literature. Fieldwork was undertaken in the United States (US), the UK and throughout the European Union (EU), and a total of 60 semi-structured interviews were conducted with a purposive sample of: US cancer patient advocates and advocates acting at the level of the supranational EU (representing 41 separate patient groups and 13 tumour types); medicines regulators; cancer specialists; journalists and other stakeholders. Ethics approval was obtained for the research and participants gave informed consent before taking part. In-depth interview guides covered a range of topics including respondents' perceptions of the therapeutic value of new drugs, patients' interests and needs, attitudes towards regulatory science and standards, the balance between evidence development and access to new medicines, patient and public participation in medicines regulation and new drug development, and the nature of relationships between the different stakeholders. Data was analysed against an initial coding frame reflecting the central research questions of the project and relevant data falling outside the initial frame was used to modify the coding iteratively, until no more useful information could be extracted.

To interrogate the plausibility of progressive accounts of medicines expansion in the treatment of patients with advanced metastatic solid tumours, this paper begins with a review of the scientific literature, including recent assessments of the clinical benefits offered by new anticancer drugs, an evaluation of these benefits in the context of studies of patient expectations and preferences, and a review of research investigating the impacts of chemotherapeutic expansion towards the end-of-life. I argue that, taken as a whole, this evidence raises significant doubts about the credibility of biomedical explanations for the increased utilisation of chemotherapy in patients with end-stage disease. I then utilise William et al.'s (2011) concept of a ‘pharmaceutical regime’ – and the shifting configuration of relationships between key actors and institutions – to explore alternative explanations for this chemotherapeutic expansion. Finally, I consider the usefulness of ‘pharmaceuticalisation’ as a lens through which to view these dynamics.

## 2 Chemotherapeutic expansion: a model of progress?

There is a lack of comprehensive and publicly accessible data on long-term, national-level trends in oncology drug prescribing. However, available data indicates that utilisation has indeed increased. According to US pharmacy benefits data analysed by Express Scripts there was a 3.4% increase in utilisation of cancer drugs between 2011 and 2012, and a 10.5% increase between 2012 and 2013 (Express Scripts, 2013, 2014). In the UK, data from one large cancer network show that between 2003/04 and 2009/10 there was a 67% increase in the number of chemotherapy courses given to cancer patients with solid tumours (Roche et al., 2010) and comprehensive, national-level data from France also demonstrates expanding utilisation of chemotherapy. Between 2005 and 2010, the number of patients receiving chemotherapy grew by 20% – faster than the growth in the number of new cancer patients diagnosed (INCa, 2012, 10). Finally, large population-based studies provide evidence of a temporal trend of increasing use of chemotherapy towards the end-of-life in North America (Earle et al., 2004, 2008; Ho et al., 2011), and the Netherlands (Bernards et al., 2013).

On first inspection, the plausibility of the biomedical thesis in accounting for this growth appears high. Globally, both the incidence and prevalence of cancer are increasing (WHO, 2013). In addition, many cancers continue to carry a poor prognosis and almost 50% of cancer patients will eventually develop metastatic disease (Koedoot et al., 2003), hence there is a desperate need for more effective treatments. Has this growing demand been matched by increased availability of effective new therapies? Here again, the indicators appear to support a progressive account of chemotherapeutic expansion. Oncology has become a major area for R&D investment by the biopharmaceutical industry over the last two decades, and this has resulted in a 70% increase in the number of drugs available to treat cancer in 2005 compared with 1995 (Analysis Group, 2013; Jonsson and Wilking, 2007). Furthermore, evaluations by medicines regulators in the US and EU indicate that between a third and one half of all new approved cancer indications offer important therapeutic advances over existing treatment options (Davis and Abraham, 2013, 155–157; Sherman et al., 2013). This would have the effect of both expanding the ‘treatable’ patient population *and* of increasing the number of drugs prescribed per patient, since new drugs are often used *in combination* with established therapies for various stages of the disease (Niraula et al., 2012; INCa, 2012, 10).

For patients with metastatic cancer, the current clinical research paradigm means that most new drugs are first tested and approved for use in patients with advanced disease who have exhausted existing treatment options (Goss et al., 2012). This may have resulted in expanded utilisation, and increased duration of chemotherapy use towards the end-of-life (Martoni et al., 2007, 420; Murillo and Koeller, 2006; Temel et al., 2008, 830; see also below). According to the industry, regulatory agencies and some research scientists and patient advocates, this ‘pharmaceuticalisation’ in the treatment of advanced cancer represents a gain for individuals and society as more patients are able to benefit from the therapeutic advances offered by these new treatments in terms of life-extension and improved quality of life (Goss et al., 2012). Yet there is accumulating evidence that whilst increasing numbers of patients with advanced disease receive drug treatment, the benefits offered by new drugs may not match patients' expectations or informed preferences and that aggressive use of chemotherapy towards the end-of-life is associated with poorer quality of life and death, regret, financial hardship and possibly shorter survival. This evidence and related issues are considered below.

## 3 What patients need and want (and what they get)

Since most metastatic solid tumours are incurable, the goal of chemotherapy for patients must be ‘palliative’ – that is, drug treatment should relieve disease-related symptoms, improve quality of life or prolong life *without* an unacceptable impact on quality (Braga, 2011; Doyle et al., 2001). Clinicians refer to care as ‘futile’ when patients are administered burdensome, toxic and potentially life threatening therapies that will not achieve any of these goals (von Gruenigen and Daly, 2005). A decision to forgo ‘disease-directed’ chemotherapy – that is, chemotherapy aimed at shrinking or stabilising the tumour – does not entail ‘doing nothing’ for patients. Instead, ‘palliative’ or hospice care focuses on relief of pain and other symptoms, and on enhancing patients' general physical, psychosocial and spiritual wellbeing (Rocque and Cleary, 2013). Whilst anticancer drugs *may* have a role to play in treating patients near the end-of-life, by definition, palliative care should not include anticancer drugs that could negatively impact on quality-of-life. The more marginal or uncertain the degree of benefit offered by drug therapy, the less likely it will be that the risk-benefit balance of drug treatment will be positive since all pharmaceutical use carries some risk.

Given evidence of chemotherapeutic expansion amongst patients with advanced disease, it is therefore particularly concerning that a number of recent, independent reviews indicate that the clinical benefit offered by the majority of new anticancer agents is at best marginal and often uncertain (Fojo et al., 2014; Garattini and Bertele, 2002; Kantarjian et al., 2013; Ocana and Tannock, 2011). Whilst it is true that a few, new anticancer drugs have genuinely changed disease prognosis and standards of care, most new drugs for advanced cancer are not in this camp (Fojo and Parkinon, 2010, 5972). For example, Sobrero and Bruzzi (2009) found that the median overall survival gain offered by new biologics over existing treatments in advanced disease was just 2 months. And with respect to the widespread belief that the new, targeted therapies are relatively non-toxic, Niraula et al. (2012) found that these agents are more likely to cause serious (grade 3–4) toxicities when administered as monotherapy or as an ‘add-on’ to standard chemotherapy regimens, and are also more likely to cause treatment discontinuation and toxic death when combined with existing regimens.

These analyses convey a very different picture to the US Food and Drug Administration's (FDA's) estimation that over half of all new oncology indications approved between 2010 and 2012 represent important advances in safety or effectiveness. If the benefits of new drugs are marginal for the majority of patients, and toxicity sometimes severe, then the risk-benefit calculation for using these drugs near the end-of-life is unlikely to be positive. This is particularly true since patients in ‘the real world’ are generally sicker, and less likely to respond positively to treatment, than patients enrolled in clinical trials (Schnipper et al., 2012; Unger et al., 2014).

There are two frequent responses to an observation that the survival gains offered by new drugs are small. First, it is sometimes claimed that the additive impact of these incremental gains may be to keep individual patients alive for significant periods of time. For example, some expert clinicians have claimed that in metastatic kidney cancer, the availability of Sutent and other targeted therapies has extended the average survival rate from 14 months to somewhere in the range of 36–48 months and that, ‘[t]argeted drugs are driving [improvements in cancer] survival in a major way’ (Wells and Pettypiece, 2010). However, recent population-based studies directly undermine such claims and show that new anticancer agents have not significantly improved cancer survival rates beyond 2–3 months (Bernards et al., 2013; Renouf et al., 2011; Saito et al., 2011; Shah and Ghimire, 2013).

Second, it is frequently asserted that cancer patients with advanced disease and short life-expectancy are willing to accept considerable toxicity for a small chance of benefit (Matsuyama et al., 2006, 3493). Here again, however, published research does not necessarily support the accepted wisdom (Mack et al., 2010). Most studies show that patients' expectations of drug therapy greatly exceed the benefits actually offered (Donovan et al., 2002; Mende et al., 2013; Silverstri et al., 1998; although see Wright et al., 2014), and that between one-third and four-fifths of patients with advanced cancer mistakenly believed that palliative chemotherapy might cure their disease (Doyle et al., 2001; Temel et al., 2011; Weeks et al., 2012). Widespread overestimation of the benefits of chemotherapy appears to influence some patients' choice to undergo treatment. For example, in a study of 134 patients with metastatic disease who had already received a median of 6-months of chemotherapy, Mende et al. (2013) found that 88% stated they would undergo treatment again. However, when asked to specify the minimum survival gain necessary for them to repeat therapy, study participants' median thresholds for survival were 18 months for non-colorectal cancer patients and 36 months for colorectal cancer patients. So while the majority of patients in this study would repeat chemotherapy, this was based on expected benefits that *far* exceed the actual survival gains offered by anticancer agents in the metastatic disease setting.

## 4 Treating patients near the end-of-life: knowing when to stop

As previously discussed, there is a lack of publicly available, comprehensive data on trends in chemotherapy use. However, in response to concerns that some patients with metastatic solid tumours may be *overtreated* with chemotherapy, a number of recent studies have investigated the extent of chemotherapy use in patients with advanced cancer. Despite considerable variation between institutions and regions, a number of studies have found high rates of utilisation near the end-of-life in both the US and the EU, along with other indicators of overuse such as administration of drugs to patients with poor performance status, chemo-insensitive tumours and several previous lines of chemotherapy (Braga et al., 2007; Colombet et al., 2012; Earle et al., 2004; Emanuel et al., 2003; Martoni et al., 2007; Murillo and Koeller, 2006; Nappa et al., 2011; Schnipper et al., 2012; Temel et al., 2008; von Gruenigen et al., 2008).

Evidence of intensive chemotherapy use near the end-of-life is not, in itself, evidence of harm. However, a number of these studies have also demonstrated that compared to patients not receiving drug treatment close to death, such treatment is associated with poorer quality of life and death – for example, a higher number of emergency room visits and admissions to ICU, fewer hospice services, decreased likelihood of patients dying in their preferred place, increased physical and psychological distress and, in some countries, bankruptcy (Earle et al., 2004; Greer et al., 2011; Mack et al., 2010; Temel et al., 2010; Wright et al., 2014; Zafar et al., 2013). Moreover, patients receiving chemotherapy near the end-of-life do not live longer than patients not receiving such therapy (Nappa et al., 2011; Saito et al., 2011; von Gruenigen et al., 2008). On the contrary, in one recent randomized study *less use* of intravenous chemotherapy was strongly associated with *increased survival* (Greer et al., 2011).

Taken as a whole, this evidence suggests that aggressive end-of-life care occurs in a significant proportion of patients in the EU and the US, is associated with poorer quality of life and death, and possibly reduced survival. Moreover, research investigating cancer patients' expectations of chemotherapy raises the possibility that a substantial number of patients with metastatic disease would *not* choose to continue drug treatment if they were genuinely made aware of its limited, or uncertain, benefits.

## 5 Understanding trends in drug treatment: the gap between rhetoric and reality

Rates of drug administration near the end-of-life vary between countries and between institutions. For example, the proportion of patients receiving chemotherapy in their final month of life in the EU ranged from 8% in one UK institution (O'Brien et al., 2006), up to 37% in a Portuguese hospital (Braga et al., 2007). Clearly, differences in national culture, the organisation and funding of healthcare – including the existence of perverse financial incentives (Keating et al., 2010) – and institutional practice are factors that will influence trends in chemotherapeutic expansion and possible overuse of drugs. However many EU studies found rates of utilization in the last 30 days of life that approached – and in some cases exceeded – those in the US, despite diverse healthcare systems and the absence of US-type financial incentives to prescribe chemotherapy. Discussions of overtreatment and futile end-of-life care in the medical literature have tended to explain these phenomena in terms of either individual physician and patient psychology, or professional culture and training (Braga, 2011; Earle, 2011; Harrington and Smith, 2008; Mack and Smith, 2012). However, these phenomena cannot explain evidence of a *temporal trend* of increasingly aggressive treatment coupled with shorter hospice stays, despite growing recognition of the importance of palliative end-of-life care and an *expansion* in the provision of palliative and hospice services.

One factor underlying the trend has been the rapid growth in the availability of drugs to treat metastatic cancer since the mid-1990s. In addition to expanded treatment options, a number of commentators have pointed to increasing pressure from patients or families as factors driving potentially futile initiation/continuation of chemotherapy (Morita et al., 2004), and recent sociological work has focused on ‘the rise of the articulate or information rich consumer’ of healthcare (Williams et al., 2011, 717). However, whilst an expanded armamentarium and growing consumerism may be proximal factors influencing recent trends, neither can adequately account for current medical practice since most new drugs offer marginal benefits which do not, according to available evidence, meet the majority of patients' *informed* preferences for, or expectations of treatment.

To understand this gap between expectations and reality we need to identify, on the one hand, key features of the current ‘pharmaceutical regime’ (Williams et al., 2011) in oncology which determine the kind of drugs we get and, on the other, the forces that are shaping patient and physician expectations. As social scientists have repeatedly demonstrated, medicines availability and use are the outcome of numerous complex and interrelated social, economic, and political (as well as techno-scientific) factors that determine how they are tested, governed, marketed and consumed. Industry, regulators, professional bodies, medical journals, the research community, expert clinicians, patient groups and the media play variously important roles in shaping the production and consumption of new drugs. The following sections provide an overview of some of these dynamics.

### 5.1 The industry-research-regulatory nexus

Industry is now the main funder of randomized controlled trials (RCTs) in oncology and this means that companies' commercial interests are the key influence in determining the objectives and design of trials (Booth, 2010). Companies seek to market their drugs and maximise profits for shareholders and investors, but in order to do this they must first demonstrate to national and supranational drug regulatory authorities that their products meet current standards of safety and efficacy. Increasingly, companies are also required to demonstrate the cost-effectiveness of their products to national health technology assessment and reimbursement bodies. Unfortunately, current standards for market approval in the US and the EU fail to ensure that new anticancer therapies offer clinically meaningful benefits patients.

First, any statistically significant difference in overall survival, however small, has been accepted as a basis for approving new drugs (Pazdur, 2008). Second, since the mid-1990s, regulators in the US and EU have further lowered the regulatory bar by introducing accelerated/conditional approval mechanisms that allow new cancer drugs to be tested against ‘non-validated surrogate endpoints’ (Davis and Abraham, 2011, 2013, 158). These surrogate endpoints – such as ‘progression-free survival’ and tumour shrinkage – are radiological measurements that demonstrate a drug has biological activity, but do *not* reliably predict whether it will improve the outcomes that really matter to patients – namely survival, symptom-control, functioning and/or quality of life (Amir et al., 2012; Booth, 2010; Booth and Eisenhauer, 2012; Fallowfield and Fleissig, 2012; Gutman et al., 2013; Salz, 2009). Permissive regulatory standards encourage companies to construct their business models and drug development programmes around small, incremental gains that create considerable uncertainty for patients, clinicians and healthcare payers regarding the true clinical value of new oncology drugs (Davis and Abraham, 2013; Fojo et al., 2014; Ocana and Tannock, 2011).

The political history and neoliberal corporate bias underlying these ‘reforms’ is described in some detail by Davis and Abraham (2013). Briefly, while changes to regulatory standards and approval mechanisms were justified on the grounds they would speed important new therapies to patients, they were in fact implemented by regulatory authorities under pressure from the US Congress and EU Commission, which were in turn driven by a determination to boost the relative economic competitiveness of their life sciences industries within the global economy (Davis and Abraham, 2011, 2013).

The current elevation of ‘international competitiveness’ by national and supranational governments as a (or perhaps ‘the’) primary concern for state policy may also be transforming the orientation of publicly-funded research in ways that are not in the best interests of patients or public health. There is evidence that public funding bodies, as well as the largest philanthropic funders, are concentrating resources on research to support drug discovery and development to the neglect of other types of investigation (European Commission, 2007; FDA, 2004; Kanavos et al., 2010). One consequence of these shifts is that it is increasingly difficult for researchers to attract funding for applied clinical research questions that do not involve drugs (Braga, 2011, 2347). This means there may now be a very poor correspondence between *both* public and private-sector research priorities and the actual concerns and information needs of patients and clinicians. For example, according to a study in which cancer patients in the UK were asked to rank their research priorities, participants were more likely to prioritise research on psychosocial issues – such as the impact of disease, how to live with it and other support issues – than research on anticancer treatments (Corner et al., 2007).

### 5.2 The oncology community

The role of the wider oncology community in supporting particular patterns of drug production and consumption in advanced cancer is mixed. On the one hand, the general culture of cancer therapy has historically been characterised by an aggressive and experimental approach to treatment (Mello and Brennan, 2001; Mukherjee, 2011) and oncologists have embraced (possibly meaningless) increments in survival (Fojo and Grady, 2009; Sacher et al., 2014). Moreover, despite acknowledgement by the oncology community of the potential for overtreatment near the end-of-life, existing guidelines from professional bodies do not specify a clear timeframe for stopping drug treatment (NCCN, 2013), and an American Society of Clinical Oncology (ASCO) panel reporting in 1996 could not decide on a minimal benefit for which chemotherapy was indicated, only that *some* proven benefit must be demonstrable (Swetz and Smith, 2010, 468).

On the other hand, some prominent clinicians have begun to focus attention on the high costs and low benefits of new cancer drugs (Bach et al., 2012; Fojo and Grady, 2009; Fojo and Parkinon, 2010; Fojo et al., 2014; Garattini and Bertele, 2002; Hall, 2013; Kantarjian et al., 2013; Niraula et al., 2012; Salz, 2009; Schrag, 2004; Seruga et al., 2010; Sobrero and Bruzzi, 2009; Sullivan et al., 2011; Zafar et al., 2013). This public questioning of the skyrocketing costs and underwhelming performance of new oncology treatments has been powerful enough to push ASCO to propose minimal efficacy thresholds for pancreatic, lung, breast and colorectal cancer (Ellis et al., 2014). However, there is by no means a consensus within the community on these issues (Berger et al., 2010; Johnson, 2014), and since companies have ultimate control over the design of the majority of clinical trials it is uncertain whether this push from within the professional community will be enough to generate change without regulatory agencies simultaneously raising standards for market approval.

The patient advocacy community has also been divided over issues of evidence, clinical value and regulatory standards. In the US, considerable disagreement exists between, on the one hand, advocates who believe current regulatory standards for the marketing of new cancer therapies are too high (acting as a barrier to both patient access and industry investment in future research) and, on the other hand, advocates who believe the standards are too low, and fail to stimulate research and development of new drugs that offer genuine therapeutic advance (Mayer, 2003; Mayer, 2010; Trowbridge and Walker, 2007).

Most patient groups rely on companies to keep them informed about new drugs in clinical trials or under regulatory review (US Patient Advocate B, 2010; US Patient Advocate E, 2011; UK Patient Advocate A, 2011; EU Patient Advocate A, 2012). But over-dependence on industry-generated data, and lack of access to alternative sources of information, means advocates may be easily persuaded to endorse a particular drug or lobby regulators for marketing approval (US Patient Advocate F, 2011), and companies may use groups to manufacture what one prominent breast cancer advocate has called ‘access advocacy’ (Mayer, 2003) where advocates pressure regulators to allow drugs to be marketed on the basis of an incomplete evidence base. Companies have also attempted to partner with groups to set up pre-marketing ‘expanded access programmes’ (EAPs) – programmes through which patients who are not eligible for clinical trials may nevertheless access an investigational product – as a way of generating support within the patient community for their drugs. EAPs, ‘access advocacy’ and patient endorsements provide companies with a means of generating expectations in the pre-marketing period and signal to investors, patients and physicians that their drug is important to patients (US Patient Advocate D, 2011; US Patient Advocate F, 2011). In countries with publicly funded healthcare systems, ‘access advocacy’ has tended to be directed at reimbursement decision-makers (Abraham, 2009, 963–964; Booth et al., 2007; Busfield, 2010, 939; Gabe et al., 2012).

Even when patient advocates are aware that a new drug offers marginal benefits, there are a number of reasons why they may be reluctant to publicly question the value of new anticancer therapies. First, some patients are genuinely willing to accept a very small chance of benefit from drug therapy and advocates feel it is their job to represent those patients' interests (UK Patient Advocate B, 2011). Advocates may also believe that patients need ‘hope’, and this belief disinclines many advocates from publicly drawing attention to the gap that can exist between rhetoric and reality (US Patient Advocate C, 2011; UK Patient Advocate C, 2012). Second, some advocates – particularly those representing patients for whom there are few, or no, good therapeutic options – value new drugs as important (albeit incremental) ‘stepping stones’ that may lead eventually to genuine breakthroughs. And advocates have described occasions when industry representatives explicitly told them that companies would not continue to develop new drugs for a specific condition if the regulatory hurdles were too high (UK Patient Advocate B, 2011). Finally, there is a real risk that companies will withdraw financial support from critical groups, and the reliance of many advocacy organisations on industry funding may contribute to an advocacy community that is unwilling to ask hard questions about new drugs (US Patient Advocate A, 2009). One advocate, for example, described how her group lost funding from two sponsors when she publicly challenged the safety of their products (US Patient Advocate D, 2011).

### 5.3 Hype and hope: (mis)representing the benefits of new drugs

In contrast to the small, incremental gains pursued by companies and sanctioned by regulators, many patients have expectations of chemotherapy far beyond the benefits that current drug treatments actually offer. One reason for this mismatch is doctors' failure to communicate honestly with patients about the real value of drug treatment (Mack and Smith, 2012), but the problem extends beyond micro-sociological factors. Within the wider ‘information landscape’ for patients, clinicians and the public, the marginal or uncertain benefits of new anticancer therapies are transformed by the alchemy of positive spin and hopeful reportage into ‘magic bullets’ and ‘miracle drugs’ (Wells and Pettypiece, 2010). Much of the information landscape is shaped by industry, but expert clinicians, research organisations, regulators, patient groups and the media play their part.

Recent studies show that unbalanced news media reporting, which exaggerates the benefits of new anticancer drugs, is the norm (Fishman et al., 2010; Hind et al., 2011) and that general reporting of cancer research overemphasises drug therapy to the exclusion of other therapeutic modalities (Lewison et al., 2008). Press releases issued by companies frequently include glowing endorsements by patient advocates and senior consultants despite minimal gains, and the news media is quick to pick up on these stories. For example, an item in the *Daily Mail,* following approval of Zaltrap for metastatic colorectal cancer, carried a claim by the UK patient group, Beating Bowel Cancer, that availability of the drug – which improved survival by 6 weeks compared to placebo – was ‘excellent news’ for patients. Further quotes include statements by senior medical oncologists that Zaltrap is ‘well tolerated’, and ‘has the potential to significantly impact survival rates in the future’ (Hope, 2013). Similarly, a US patient advocacy group, Colon Cancer Alliance, reported on its website that recent approvals of Zaltrap and another drug, marked ‘a milestone in the treatment of colon cancer’ (Colon Cancer Alliance, undated). In contrast to these public pronouncements, an experienced and knowledgeable patient advocate in the US privately stated that Zaltrap was associated with serious (grades 3–4) toxicities and did not represent a meaningful advance for patients (US Patient Advocate D, 2011).

Regulators also generate unrealistic expectations, both in relation to the current benefits offered by new therapies and in relation to the promise of future advances. For example, the Commissioner of the FDA recently proclaimed that, ‘America is at an important crossroads, where the science before us presents unprecedented opportunities to create new and better medical products and to promote better health for the public’ (FDA, 2011). In a similar vein, though with slightly less fanfare, a senior FDA scientist reassured the public that the new anticancer drugs under agency review were ‘slam dunks. It's not *if* we're going to approve them. It's how fast we're going to approve them’ (Harper, 2013).

As well as exaggerating the benefits of new drug treatments, public discourse about cancer research is overwhelmingly positive and *hopeful*. Cancer is the ‘poster child’ for scientific advances in the molecular understanding of disease, and is constantly cited as the field in which the promise of personalised medicine is closest to being realized (Schilsky, 2010). This hopeful reporting of the state of cancer research and medicines originates from individual researchers, and non-profit and for-profit institutions alike (Anon, 2000, 157), and may explain why the majority of people believe that a cure for cancer will either ‘definitely’ or ‘probably’ be found within 50 years (Anon, 2010).

Rarely does the public get to hear beyond the drumbeat of medical progress. Even when patients and clinicians turn to the scientific literature for reliable information, they may still be misled over the value of new anticancer therapies. New drugs for non-small-cell lung cancer (NSCLC) offering an additional 5 weeks of survival were reported in ASCO press briefings as drugs ‘likely to have a significant impact on the care of patients’ (Fojo and Parkinon, 2010, 5973). As with other therapeutic areas, numerous analyses have found evidence of biased and incomplete reporting of trial results in the scientific and promotional literature (Altwairgi et al., 2012; Rasmussen et al., 2009; Vera-Badillo et al., 2013; Wick et al., 2007), and the ‘positive’ interpretation of oncology trials has increased, along with the misreporting of trial results, over the period in which industry replaced government as the main funder of research (Booth, 2010; Sacher et al., 2014). Publication bias, distorted scientific reporting, promotional material, and stories of ‘miracle’ drugs percolate against a background discourse of ‘science at a crossroads’ and ‘new eras’.

## 6 Conclusion

This article explores trends in chemotherapeutic expansion amongst patients with advanced, metastatic solid tumours. While some of this growth can undoubtedly be explained by an account of medicines consumption in which pharmacological progress and improved care meet expanding health needs – there is also evidence of inappropriate and overly aggressive use of drugs. This evidence demonstrates that although an increasing number of patients with advanced disease receive drug treatment, this treatment may not match their subjective expectations or informed preferences and that, irrespective of patient preferences, aggressive chemotherapeutic treatment towards the end-of-life is associated with poorer quality of life and death, shorter survival, regret, and in some cases severe financial hardship.

Studies in the medical and psychological literature have attempted to account for over-use in terms of individual patient and physician behaviour and psychology. By situating chemotherapeutic expansion within the conceptual framework of a wider ‘pharmaceutical regime’ we are able to trace the broader cultural, political and institutional influences on patterns of drug production and consumption. This analysis confirms the pivotal roles played by governments, the medical and research communities, regulatory bodies, patient groups, and the media (Abraham, 2009, 2010; Busfield, 2010; Williams et al., 2011). Further, it shows that pharmaceutical companies' control over the organisation and funding of research, it's ability to shape the information landscape, and the prioritisation of industry interests within regulatory agencies and at the political level more broadly may entail negative consequences for patients, public health and society as a whole.

First, excessive hype surrounding new anticancer therapies creates a gap between what patients and the public *expect* from drug therapy and what current regulatory standards, research, treatment norms and industry business models deliver. This has helped to fuel chemotherapeutic expansion and overtreatment of patients near the end-of-life, with its attendant risks of physical, psychological and financial harm. Second, given the marginal or uncertain benefits of many new anticancer drugs, significant societal resources have been (and continue to be) expended on research and healthcare in a way that may be suboptimal for current and future patients' health (Fojo et al., 2014). Third, current regimes for funding clinical research have generated an excessive focus on pharmacotherapy in the care of cancer that may increasingly crowd out the pursuit and funding of research and healthcare options not involving drugs (Braga, 2011; Kanavos et al., 2010; Lewison et al., 2008) – an example, perhaps, of the colonisation of health futures referred to by Williams et al. (2011, 719–729). This is not to say that patients do not benefit from and value drug treatments, or to deny that there is a real and urgent need for genuine therapeutic breakthroughs that will significantly improve health outcomes. It is simply to acknowledge that patients have psychosocial, health, and support needs (including the need to come to terms with death) which go beyond and sometimes outweigh the need for new anticancer treatments (Corner et al., 2007; Fallowfield and Jenkins, 2013).

Whilst confirming the value of an analytic framework that explores the relationships between multiple upstream and downstream factors in the production and consumption of anticancer drugs, there are nevertheless issues raised by this case study that are not adequately addressed by the concept of pharmaceuticalization as currently defined and which point, I believe, to the need for further clarification and justification of pharmaceuticalization's conceptual boundaries.

Both Abraham (2009, 604–5), and Williams et al. (2011, 710–11), imply that the process of pharmaceuticalization involves some *re-designation* of a condition as one suitable for pharmaceutical intervention with new or existing drugs. It could be argued that the documented trend towards increasingly aggressive and late treatment of cancer patients near the end-of-life falls within this definition. But there are instances of increased chemotherapeutic consumption that are not obviously cases of pharmaceuticalization since they involve stages of the disease that were already deemed suitable for drug treatment. For example, new drug treatments are often *added* to an existing regimen of one to two chemotherapy agents, which generally also adds to the toxicity of treatment (Niraula et al., 2012). Such an expansion in medicines use is the product of the same ‘socio-technical regime’ (Williams et al., 2011) and may carry similar consequences for patients and society, so it is unclear why the former would be conceptualised as ‘pharmaceuticalisation’, but not the latter.

We might resolve this issue by suggesting that ‘pharmaceuticalisation studies’ should encompass *any* instance of medicines expansion (or decline) in use. Yet here again, there does not appear to be a clear intellectual justification for privileging a focus on cases involving increasing/decreasing medicines production and consumption, particularly if, as social scientists, we are interested in evaluating both the ‘normative raison d'etre’ of healthcare and the implications of medicines use for patient and public health (Timmermans and Haas, 2008). For example, aggressive industry marketing tactics that result in the substitution of new, more expensive (but possibly less effective and more toxic) drugs for cheaper generics is as important to understand and document as disease-mongering or off-label use (Gale, 2001; Hill et al., 2008; Light, 2010, 12–13). Such practices may or may not lead to an increase in the number of pills consumed per head of population, but they can still fuel the inappropriate and harmful utilisation of medicines.
